# Baisse de l'acuité visuelle majeure révélant un mélanome choroidien malin

**DOI:** 10.11604/pamj.2014.17.277.4217

**Published:** 2014-04-14

**Authors:** Rajae Derrar, Rajae Daoudi

**Affiliations:** 1 Service d'ophtalmologie A, Hôpital des spécialités, CHU Université Mohammed V Souissi, Rabat, Maroc

**Keywords:** Acuité visuelle, mélanome choroidien, visual acuity, choroidal melanoma

## Image en medicine

Le mélanome choroïdien constitue la tumeur oculaire primitive la plus fréquente et la plus redoutée de l'adulte. Nous présentons le cas d'une patiente âgée de 45 ans ayant consulté pour une baisse d'acuité visuelle d'installation rapidement progressive. L'examen trouve une acuité limitée à perception lumineuse positive avec un examen du segment antérieur normal, l'examen du segment postérieur montre une masse sous rétinienne achromique convexe dans le vitré, le tonus oculaire était à 10 mmHg L'angiographie montre une hyperfluorescence aux temps précoces, intermédiaires et tardifs avec aspect de pin point. Une échographie oculaire, une imagerie par résonance magnétique ainsi qu'une angiographie ont été demandé, une échographie hépatique à montré l'absence de métastases. Un avis oncologique a été demandé et la patiente à subit une énucléation. L’étude anatomopathologique a confirmé le haut degré de malignité du mélanome. Le mélanome malin est une tumeur fortement redouté, son traitement varie en fonction du degré d’évolution au moment de découverte, la radiothérapie, curiethérapie, hadronthérapie permettent d’éviter l’énucléation

**Figure 1:**
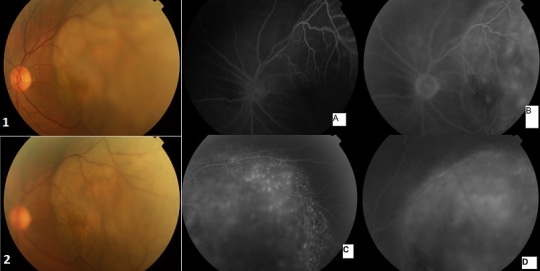
Masse sous rétinnienne convexe dans le vitré (1,2); angiographie à la fluorescéine montrant une hyperfluorescence aux temps précoces (A), intermédiaires(B) et tardifs(D) avec aspect en pin point (C)

